# Arginase induction and activation during ischemia and reperfusion and functional consequences for the heart

**DOI:** 10.3389/fphys.2015.00065

**Published:** 2015-03-11

**Authors:** Klaus-Dieter Schlüter, Rainer Schulz, Rolf Schreckenberg

**Affiliations:** Physiologisches Institut, Justus-Liebig-Univiersität GiessenGiessen, Germany

**Keywords:** ornithine, nitric oxide, polyamines, reactive oxygen species, reperfusion injury

## Abstract

Induction and activation of arginase is among the fastest responses of the heart to ischemic events. Induction of arginase expression and enzyme activation under ischemic conditions shifts arginine consumption from nitric oxide formation (NO) to the formation of ornithine and urea. In the heart such a switch in substrate utilization reduces the impact of the NO/cGMP-pathway on cardiac function that requires intact electromechanical coupling but at the same time it induces ornithine-dependent pathways such as the polyamine metabolism. Both effects significantly reduce the recovery of heart function during reperfusion and thereby limits the success of reperfusion strategies. In this context, changes in arginine consumption trigger cardiac remodeling in an unfavorable way and increases the risk of arrhythmia, specifically in the initial post-ischemic period in which arginase activity is dominating. However, during the entire ischemic period arginase activation might be a meaningful adaptation that is specifically relevant for reperfusion following prolonged ischemic periods. Therefore, a precise understanding about the underlying mechanism that leads to arginase induction as well as of it's mechanistic impact on post-ischemic hearts is required for optimizing reperfusion strategies. In this review we will summarize our current understanding of these processes and give an outlook about possible treatment options for the future.

## Arginase isoforms and biological activity

Arginase is an enzyme that catalyzes the hydrolysis of L-arginine into urea and ornithine. It is expressed in two different isoforms. Arginase I is located in the cytosol and well-known from hepatic metabolism in which arginase is responsible for the elimination of metabolites from amino acid and nucleotide metabolism. The enzyme is expressed throughout the body in endothelial cells and muscle cells (Gonon et al., 2012). In contrast to arginase I, arginase II is predominantly located in the mitochondrial matrix and seems to be linked directly to polyamine metabolism, although its exact function is not known (Morris, 2005). The polyamine metabolism synthesizes polyamines such as spermine and spermidine that are required for cellular growth. The rate limiting step of this pathway is the induction of ornithine decarboxylase (ODC) converting ornithine into putrescine. Polyamines, such as putrescine, spermine, and spermidine, may act within cells but they can also be released from cells. In the latter case they affect cell function as agonists of calcium-sensing receptors (Smajilovic et al., 2010). Arginase II may not be confined to the mitochondrial matrix when the expression level is elevated. Moreover, arginase can also be found in platelets where it plays a role in sickle cell disease not necessarily requiring mitochondrial participation (Raghavachari et al., 2007). When not specified within the text, the term arginase will be used as a synonym for both isoforms.

Arginine as the main substrate of both isoforms of arginase is also the substrate for the three isoforms of nitric oxide synthase (NOS). Therefore, excessive activity of either NOS or arginase reduces substrate availability for the other arginine consumer. In fact, most cells express arginase and NOS isoforms at the same time. In most cells enzymatic activity of each of these enzymes is tightly regulated by direct protein modification or by induction of enzyme expression. In case of arginase an increased activity of arginase has consequences for both pathways: a loss of NO/cGMP signaling and an improvement of polyamine metabolism. Examples for increased arginase activity and expression are found in artherosclerosis, hypertension, inflammation, aging, stroke, myocardial infarction, and heart failure to name just a few (Wu and Meininger, 2000; Ryoo et al., 2008; Bagnost et al., 2010; Heusch et al., 2010). It is commonly accepted that excessive activation of arginase is associated with disease progress. Whether the final effect of arginase activation depends on the activation of arginase-dependent pathways (polyamine pathways) or on the inhibitory effect on NO/cGMP-dependent pathways or on both pathways remains elusive. Furthermore, at least in case of atherosclerosis, arginase II may activate intracellular signaling independent of its enzymatic activity (Xiong et al., 2013). Regarding the role of arginase in endothelial dysfunction, arginine metabolism seems to play a relevant role by either reducing arginine uptake (Martens et al., 2014), activation of arginase II (Pandey et al., 2014), or activation of arginase I (Gao et al., 2007). A precise understanding of the regulation and function of arginase and a subsequent translation of these findings into medical therapies will certainly improve clinical outcomes. The current review will focus on the role of arginase during ischemia and in particular during reperfusion.

## Arginase activation in ischemic and post-ischemic hearts

**Figure 1:**
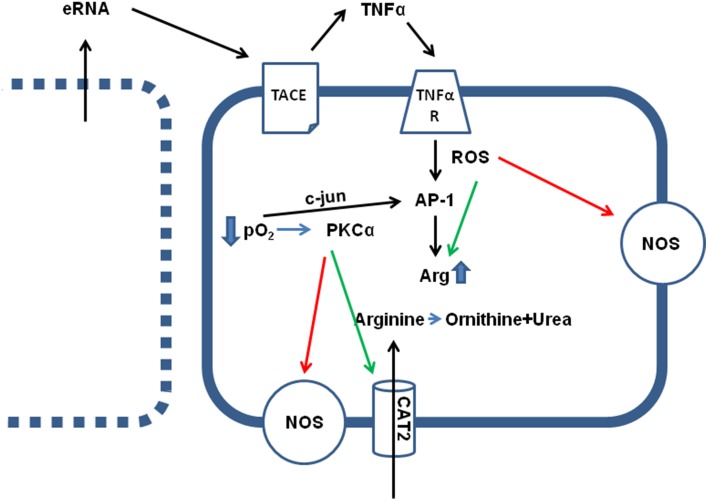
**Effect of hypoxia (ischemia) on arginine metabolism**. Direct effects of low pO_2_ are the translocation of c-jun in AP-1 dimers, thereby activating AP-1 transcriptional activity, an activation of PKCα and subsequent activation of arginine transporters (CAT2) and inhibition of constitutively expressed NOS isoforms (eNOS and nNOS). Indirect effects of low pO_2_ are loss of sarcolemmal integrity in some cells (see left), leading to the release of intracellular particles, such as RNA (eRNA), that activates a sheddase (TACE) thereby releasing TNFα. TNFα augments the hypoxia-induced cell damage by activation of AP-1 and increasing arginase (Arg) expression, by activation of arginase activity via nitrosylation and reducing the K_M_ value for the enzymatic reaction, and ROS-dependent inhibition of NOS activity.

Most, but not all investigators that studied the activity of arginase in ischemic and post-ischemic cardiac tissues found a significant increase in total arginase activity, arginase I expression, or both. In principle, expression of arginase II can also be induced by hypoxia as shown for human pulmonary artery smooth muscle cells (Chen et al., 2014; Jin et al., 2014). However, whether induction of arginase II contributes to the increased arginase activity in the heart during ischemia and reperfusion remains to be established. Most remarkable observations of increased arginase activity were made at a very early time-point after the onset of ischemia and reperfusion (Harpster et al., 2006; Grönros et al., 2013). The underlying mechanism by which arginase I expression is induced has been evaluated in detail as described now. Arginase I expression was identified as the strongest and fastest transcriptional adaptation during ischemia and reperfusion in the heart (Harpster et al., 2006). Several mechanisms seemed to be responsible for this effect: At first, hypoxia and reoxygenation damages cardiomyocytes because it leads to excessive calcium load during ischemia and subsequent reoxygenation generates energy that allows a very strong contraction disrupting the sarcolemmal membrane. This cell damage leads to a release of intracellular material into the extracellular compartment (Hearse et al., 1973). This hypercontraction-induced cell damage is the basis for diagnosis of infarct size by quantification of plasma levels of cardiac-specific enzymes, i.e., hs cTnI (= high sensitive cardiac-specific troponin I). Such molecules that are released by hypercontracture may have also a functional relevance in the subsequent activation of arginase expression. Extracellular RNA (eRNA), which is among intracellular materials released by hypercontracture, triggers the activation of a membrane-bound sheddase that, once activated, releases TNF-α (Cabrera-Fuentes et al., 2014; see Figure 1). TNF-α has been identified as a pro-inflammatory cytokine that activates arginase I (Schreckenberg et al., 2015b). This observation is further based on experiments with TNF-α^−/−^ mice in which ischemia and reperfusion does not lead to an induction of arginase I (Gao et al., 2007). TNF-α may trigger this process via activation of the transcription factor AP-1 (Figure 1). A potential AP-1 binding site has been identified in the promoter region of arginase and TNF-α activates the activity of the transcription factor AP-1 (Zhu et al., 2010). Hypoxia directly recruits c-jun to AP-1 binding sites of the arginase-1 promoter (Singh et al., 2014). c-Jun binds together with activating transcription factor-2 at the AP-1 site, which initiates the transactivation (Zhu et al., 2010). Acute myocardial infarction is sufficient to induce the expression of 15 different genes that are involved in assembly and activation of AP-1 within 15 min (Harpster et al., 2006). All these findings strongly support the assumption that AP-1 activation plays a major role in adaptation of arginine metabolism to hypoxia. Such a scenario would also explain the more general finding that arginase activation under ischemia and reperfusion is not specific for the heart but represents a more general pathway by which tissues respond to hypoxia because cell damage and loss of plasmalemmal integrity is a characteristic feature of anoxic cell damage. As outlined in the next section, hypoxic conditions trigger arginase I expression in nearly all tissues. As mentioned above, arginase II can also be induced by hypoxia. The mechanism has been worked out on pulmonary smooth muscle cells. Hypoxia induced the expression of miR-17-5p that then triggers the up-regulation of arginase II (Jin et al., 2014). Activation of PI3-kinase-Akt signaling pathways can attenuate this activation (Chen et al., 2014). However, it remains to be clarified whether similar concerns hold for arginase II in the heart during acute ischemia and reperfusion.

At least within cardiac tissue, induction of arginase I expression is a strong and early response of cells to hypoxic stress. This leads to the question whether this is exerts a protective effect? At first, during the hypoxic period the arginine-consuming counterpart of arginase, NOS, is functionally inactive, because formation of NO from arginine requires molecular O_2_. In the absence of O_2_, NOS works in an uncoupled mode generating reactive oxygen species (ROS) that damages cells and this may be relevant at the onset of reperfusion. In this context it is also likely to understand that ROS stimulates arginase expression as well (Figure 1). Moreover, TNF-α activates ROS formation. In other words, ROS formation initiates a negative feedback loop by which arginine, the substrate of NOS, is removed in a condition in which uncoupled NOS forms damaging ROS.

**Figure 2:**
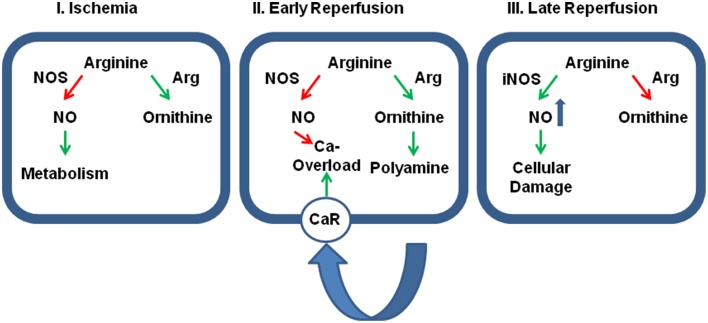
**Relative contribution of cellular protection vs. cellular damage of the two main arginine pathways. Panel (I)**: During ischemia the activation of arginase (Arg) activity reduces the effect of physiological levels of NO on cellular metabolism thereby and reduces otherwise accumulating arginine levels. At this time, inhibition of arginase would be detrimental for the outcome. **Panel** **(II)**: During early reperfusion, dominance of arginine pathway limits the protective role of NO, specifically on SR-calcium load that triggers fatal arrhythmic events. Moreover, the activated arginase/polyamine pathway potentially augments calcium overload by release polyamines that act on calcium receptors (CaR). Now, the further outcome will benefit from arginase inhibition. **Panel** **(III)**: At late reperfusion, inflammatory pathways have induced the expression of iNOS that generates detrimental amount of NO that are potentially damaging. In this phase, an inhibition of arginase will be again detrimental, because then more arginine can be converted into NO. Green arrows indicate activation and red arrows indicate inhibition.

Activation of arginase cannot reduce the level of overburden ROS formation alone. In transgenic mice with over-expression of TNF-α, it was found that the subsequent development of endothelial dysfunction can be reduced by administration of tempol, a superoxide dismutase (SOD) mimetic and radical scavenger (Zhang et al., 2010). In chronic ischemic injury, up-regulation of arginase I and SOD-2 are commonly found and it remains speculative whether the up-regulation of SOD-2 is not sufficient for ROS scavenging or whether arginase I activation and SOD-2 activation play divergent roles (Roy et al., 2009). Another piece of this puzzle is the finding that increased ROS formation favors S-nitrosylation of arginase thereby decreasing the K_m_ for L-arginine again favoring the reduction of arginine levels. Furthermore, activation of protein kinase C (PKC)-α in endothelial cells further decreases the activity of NOS by activation of the transcription factor AP-1 that induces not only the expression of arginase but also that of the up-take of arginine by induction of the expression of CAT-2 transporters (Figure 1). At the same time, PKC-α promotes eNOS phosphorylation at Thr 495 resulting in decreased NO production (Visigalli et al., 2010). Finally, in the ischemic/reperfused heart, arginase I induction is opposed by eNOS down-regulation (Hein et al., 2003). All these molecular responses to hypoxia shift arginine consumption from the NO pathway toward the arginase-dependent pathways and this may be a strategy of cells to withstand hypoxic stress. In conclusion, under conditions in which NOS cannot synthesize NO, reducing the intracellular arginine pool by arginase activation may protect cells. However, high concentrations of arginine can reduce the expression of NOS which is then lacking in the reperfused tissue. All these examples suggest that reducing arginine levels will protect hypoxic cells from irreversible damage (Figure 2-I).

This advantage of high arginase activity during ischemia may switch into a strong disadvantage as soon as the tissue is reperfused. Now, high arginase activity contributes to the reduced NOS activity in post-ischemic tissues resulting in low perfusion and prolongation of ischemic time. Moreover, low NO/cGMP levels contribute to reperfusion injury, loss of endothelial barrier function, and increase the susceptibility to cardiac arrhythmia (see Section Post-Ischemic Consequences of Reduced NO Formation and Contribution of Arginase for details). At the same time, the release of polyamines acts via activating of calcium-sensing receptors and this affects the rather sensitive intracellular calcium handling in cells during reperfusion (Figure 2-II).

## Arginase activation in non-cardiac tissues

As mentioned above, arginase activation during hypoxia is not tissue specific. Like the heart, arginase activation occurs in the liver and some other tissues such as the detrusor (Kawano et al., 2006). Although this review is focussed on the heart, it seems to be important to compare the effect of ischemia on arginase activity in the heart with that in other organs to better understand the relevance of arginase activity in cells. Investigating the effect of ischemia on arginase activity was first performed in the liver. In the liver arginase is the most important enzyme that protects the organism from ammonia intoxication. Excessive release of arginase from liver transplants is a severe clinical problem that occurs in the reperfusion period. Arginase is able to metabolite plasma arginine and thereby reduce the substrate availability for NOS and arginase. Subsequently, a drop in plasma arginine and nitrite, a stable end product of NO, and an increase in plasma ornithine can be observed. Functionally, this leads to hemodynamic alterations specifically in the lung and liver (Roth et al., 1994; Längle et al., 1995; Silva et al., 2005). Supplementation of arginine could reduce liver transplant preservation injury in rats with orthotopic liver transplantation (Yagnik et al., 2002). Vice versa, inhibition of arginase by a specific inhibitor of arginase, Nor-NOHA, stabilized liver histology and function (Reid et al., 2007; Jeyabalan et al., 2008). A stabilization of arginase activity could be achieved by ischemic pre-conditioning of the liver (Ofluoglu et al., 2006). Moreover, administration of adrenomedullin, a multifunctional peptide with a putative beneficial role after ischemic insults, normalized arginase activity and NO formation. Interestingly, it reduced also the induction of the pro-inflammatory TNF-α pathway (Kerem et al., 2008). As outlined already, TNF-α is a likely candidate for arginase induction. Collectively, the data show a strong induction of arginase activity in ischemic/reperfused liver tissue that leads to a substrate deficit for NOS and thereby contributes to the post-ischemic tissue damage. Therefore, the situation in the liver is similar to that of the heart and supports translation of these findings to heart protection against ischemia and reperfusion injury.

On the other hand, reducing arginine pools by hyperactivation of arginase will also have beneficial effects as it limits the activity of iNOS in macrophages (Figure 2-III). Indeed pro-inflammatory macrophages (M1 cells) express large amounts of iNOS while anti-inflammatory macrophages that are involved in wound healing (M2 cells) express large amounts of arginase. Unfortunately, M2 macrophages are not only responsible for wound healing as for example in post-ischemic tissues but also for tumor growth. The relationship between M1 and M2 macrophages can be triggered by a specific tyrosine kinase, namely the Ron receptor tyrosine kinase, that upregulates Fos and enhances binding of Fos to the AP-1 binding site that has already been identified as a promoter element required for arginase I induction (Sharda et al., 2011). M2 macrophages play a distinctive role in reperfusion injury of the kidney. In the kidney M2 macrophages trigger the repair process days after the ischemic insult (Lee et al., 2011). Indeed, the role of arginase activation in post-ischemic tissue differs between the kidney on the one hand and the liver and heart on the other hand. Whereas, arginase is activated in liver and heart, arginase activity is reduced in post-ischemic kidneys. In the kidney increased NO production leads to augmented tubular injury. Hypoxia leads to an up-regulation of CAT-2 thereby providing arginine as a substrate for eNOS and iNOS (Schwartz et al., 2002). In addition, arginase I and arginase II expression is reduced in post-ischemic kidney in contrast to the liver and heart. Erbas et al. showed that administration of N-acetylcysteine normalizes arginase activity in the kidney and improved the situation following ischemia and reperfusion (Erbas et al., 2004). Noteworthy, carefully measurements of renal arginase activity 2 weeks after the insult have revealed an up-regulation of the enzyme in the kidney that is required for the proliferative repair of the damaged kidney (Bogatzki, 1963). As in the kidney, ROS in the presence of high NOS activity trigger neurological disorders to hypoxia/anoxia in brain and improving arginase activity is again associated with protection (Swamy et al., 2010).

Collectively these data show different roles for arginase in hypoxic/reoxygenated tissues and indicate that a simple relationship between tissue damage and arginase activity is unlikely to be identified. Concerning the consequences of arginase activation in the heart, we may conclude from these studies on non-cardiac tissues that arginase activity affects different physiological activities in post-ischemic tissues that are linked to wound healing, tissue repair, and cell function. The data suggest that arginase activation may be necessary for some post-ischemic physiological adaptation but may also be detrimental for other ones. Like the kidney pharmacological options to affect the arginase activity will need to define the best time-window to either improve or inhibit activation. The consequences for arginase activity in the heart will be mentioned in the next section.

## Post-ischemic consequences of reduced NO formation and contribution of arginase

As outlined above, arginase activation increases the formation of ornithine but also limits the substrate availability for NOS. Substrate limitation causes reduced NOS activity and NO has repeatedly shown before to be important for proper heart function (Rassaf et al., 2006). Reduced NOS activity may reduce ROS formation during ischemia but low NO levels are a common problem in post-ischemic hearts. NOS activity in endothelial cells is required for proper perfusion of the heart and as well as for the anti-thrombotic activity of NO. Impaired endothelial function in post-ischemic hearts could be restored by inhibition of arginase activity as repeatedly been shown (Hein et al., 2003; Gao et al., 2007; Grönros et al., 2013). Furthermore, NOS activity is also important within cardiomyocytes where NO/cGMP signaling contributes to calcium oscillation during the phase of reperfusion, although this may also be responsible for cell damage and arrhythmia. Indeed cardiac de-synchronization is a major problem in the early phase of reperfusion. NO/cGMP is required for normal cardiac function as it regulates calcium handling, ion channel open probability and thereby action potential duration and other aspects of cardiac function. Among them are effects on cellular cAMP levels by cGMP-dependent inhibition of phosphodiesterase (PDE) II, protein kinase G-dependent down-regulation of voltage-dependent L-type Ca^2+^ currents, desensitization of cardiac myofilaments by phosphorylation of troponin I, and metabolic effects. These consequences of reduced NO in cardiomyocytes are reviewed by Massion et al. (2005). Despite clear effects of cGMP on calcium currents, NO/cGMP also modulate ATP-sensitive K^+^ channels, the hyperpolarization-activated pacemaker current I_f_, and voltage-dependent fast Na^+^ currents (Fischmeister et al., 2005). Furthermore, while NO/cGMP pathways inhibit cardiac hypertrophy, an activation of arginase-dependent polyamine metabolism is pro-hypertrophic (Schlüter et al., 2000; Booz, 2005). As expected some of these stressors could be minimized by improving NO/cGMP signaling in post-ischemic hearts. Moreover, uncoupling of NOS caused by hypoxia increases ROS formation and excessive ROS, due to oxidative stress, directly impairs cardiac function by formation of disulfides cross-bridges at tropomyosin (Canton et al., 2006). As a consequence arginase activation in post-ischemic hearts may have detrimental effects due to the substrate limitation of NOS and may contribute to NOS inefficiency at that time points.

Although increased substrate availability of arginine during reperfusion may allow NOS to generate NO/cGMP, the interaction between arginase and NOS is more complex than perceived. Indeed, a clinical trial that was aimed to increase substrate availability by administration of arginine to post-infarct patients had to be stopped due to lack of benefit and, even more important, risk of increased mortality (Schulman et al., 2006). These data indicate a detrimental effect of arginase-dependent pathways in the post-ischemic period. As the polyamine metabolism is one of the major pathways activated by arginase activity we will discuss the consequences of polyamine metabolism for the post-ischemic heart next.

## Post-ischemic consequences of increased polyamine metabolism and contribution of arginase

Arginase activation not only limits the activity of NOS isoforms but also generates the substrate for the polyamine metabolism of ornithine. An activation of the polyamine metabolism is normally associated with anabolism. In terms of cardiac tissue, activation of polyamine metabolism is required for cardiac hypertrophy. An activation of the polyamine metabolism critically depends on the induction of ODC, the rate limiting enzyme of the polyamine metabolism. Therefore, activation of arginase and ODC creates a situation in which synthesis of polyamines is most likely. The polyamine metabolism in general must be considered as a meaningful adaptive mechanism because it allows cardiac hypertrophy that means that survived cardiomyocytes can increase the number of their contractile units, the sarcomers. Furthermore, release of polyamines can initiate an autocrine loop that activates calcium-sensing receptors on cardiomyocytes that by itself increase cardiac power (Schreckenberg et al., 2015a). However, as often in physiology it is difficult to adapt the right power of activation. If the activation of the polyamine metabolism is excessive the same pathway favors the induction of apoptosis (Giordano et al., 2010; Mörlein et al., 2010). Moreover, as activation of calcium-sensing receptors activates a Gαq-dependent signaling in cardiomyocytes this involves the activation of IP_3_ receptors that trigger again arrhythmic events (Schreckenberg et al., 2015a). Collectively, activation of arginase in the post-ischemic myocardium has an increased risk of mal-adaption.

## Experiences with arginase inhibition in ischemic and reperfused hearts

As outlined above, a successful use of arginase inhibitors to improve post-ischemic recovery is limited at present by lack of knowledge about exact time, duration, and amount of inhibition. Nevertheless, recent studies have mostly shown beneficial effects if arginase inhibition was performed during the early phase of reperfusion. The majority of these studies have revealed that arginase inhibition significantly improves NO production in post-ischemic hearts (Jung et al., 2010; Gonon et al., 2012; Tratsiakovich et al., 2013). Moreover, inhibition of arginase before reperfusion reduced infarct sizes via preserved NO production (Grönros et al., 2013). Another potential mechanism by which inhibition of arginase during or immediately after reperfusion reduces reperfusion injury lies in the activation of PKC-ε and opening of mitochondrial ATP-dependent K^+^ channels (mitoK_ATP_). However, it remains unclear whether this already clarifies the role of arginase during reperfusion injury of the heart. Pharmacological activation of PKC-ε failed to improve strong end-points such as death and heart failure albeit reduction in infarct size (Mochly-Rosen et al., 2012). Furthermore, dietary supplementation of arginine in post-infarct patients to attenuate arginase-induced arginine depletion in target cells turned out to be not effective (Schulman et al., 2006). Even more disappointing, this trial had been stopped for increased mortality in the treatment group. Promising experimental data show a reduction in reperfusion injury by inhibition of arginase. However, up-regulation of arginase-2 in erythropoietin-treated rat hearts showed improvements in contractility and reduced myocardial (Lu et al., 2012). In a recent study on isolated rat hearts, Yang and colleagues provided evidence that inhibition of arginase I activity in red blood cells increases NO release from erythrocytes thereby improving post-ischemic recovery (Yang et al., 2013). While it is easily to understand that increased NO may improve post-ischemic recovery, it is difficult to explain why the inhibition of tissue-specific arginase activation does not play a role in these hearts unlike the inhibition of arginase in erythrocytes. Finally, in the setting of chronic ischemia, arginase activity is not related to coronary microvascular dysfunction (Sodha et al., 2008). Collectively, it remains unclear whether the hypothesis of substrate limitation is really cause of arginase-dependent malfunction in post-ischemic hearts and how post-ischemic hearts benefit mostly from arginase inhibition.

## Possible targets to modify arginase activity and the consequences of arginase activation in post-ischemic hearts

Most studies published to date have used the arginase-specific inhibitor N^ω^ -hydroxy-nor-L-arginine (Nor-NOHA) to inhibit arginase during reperfusion. Nor-NOHA has the advantage not to interfere with NOS. Most but not all data published to date are promising (see above) but it remains unclear whether treatment with an arginase subtype unspecific inhibitor is optimal. Consequently, the development of other arginase inhibitors underway. Congeners of (R)-2-Amino-6-borono-2-(2-(piperidin-1-yl)ethyl)hexanoic acid have been shown to bind preferentially to arginase I, have antagonistic effects and are orally bioavailable (Van Zandt et al., 2013). Such compounds may be used to identify isoform specific effects of arginase in the future. Another problem might be the timing of inhibition. Arginase activation may be required during ischemic insults, detrimental during the early phase of reperfusion but again required at later time points to shift macrophage activity from pro-inflammatory M1 to anti-inflammatory M2 subpopulation. Long-time follow-ups are required to define electrophysiological remodeling and such data are not yet reported. As indicated in case of PKC-ε, reduction of infarct size alone is not sufficient. Another point that has to be clarified in the future is whether arginase inhibition is optimal or whether upstream elements of arginase induction are more effective. Alternatively to arginase inhibition an inhibition of TNF-α activation that attenuates arginase I activation might be more effective. TNF-α knock-out mice had no up-regulation of arginase. Subsequently, inhibition of a sheddase that releases bound TNF-α during hypoxia was sufficient to attenuate arginase induction (Schreckenberg et al., 2015b). Unlike the direct inhibition of arginase, such an approach does not block arginase activity in M2 macrophages. Therefore, a better understanding of the mechanism by which arginase is activated and contributes to reperfusion injury will lead to more specific targets to interfere with these pathways. If substrate competition between NOS and arginase is the main problem at the time of reperfusion, citrulline rather than arginine supplementation may be of specific advantage. Citrulline has less side effects, can be better administrated orally than arginine, because it is not metabolized in the liver, and can be converted into arginine at the site of action within the cell due to the close physical association between argininosuccinate lyase and eNOS (Romero et al., 2006; Smith et al., 2006).

## Conclusive remarks

Arginase activation is a common event in hypoxic tissues including the heart. Recent development of reperfusion therapies has led to a situation in which high arginase activity at the onset of reperfusion participates in reperfusion-induced damage. Therefore, there seems to be a therapeutic window by which either attenuation of arginase induction of inhibition of arginase activity attenuates reperfusion damage. The optimal timing of pharmacological interference has still to be defined as well as the optimal time point. Figure 2 summarizes these assumptions.

### Conflict of interest statement

The authors declare that the research was conducted in the absence of any commercial or financial relationships that could be construed as a potential conflict of interest.

## Abbreviations

NOS: nitric oxide synthase
TNF: tumor necrosis factor
ODC: ornithine decarboxylase
ROS: reactive oxygen species
SOD: superoxide dismutase
Nor-NOHA: N^ω^ -hydroxy-nor-L-arginine.
